# Attitudes of COPD Patients towards Tele-Rehabilitation: A Cross-Sector Case Study

**DOI:** 10.3390/ijerph10116184

**Published:** 2013-11-15

**Authors:** Birthe Dinesen, Lotte Huniche, Egon Toft

**Affiliations:** 1 Telehealth Laboratory, Integrative Neuroscience Research Group, Center for Sensory-Motor Interaction, Department of Health Science and Technology, Faculty of Medicine, Aalborg University, Fredrik Bajers Vej 7 D3, Aalborg DK-9220, Denmark; 2 Institute of Public Health, University of Southern Denmark, J.B. Winsloews Vej 9, Odense DK-5000, Denmark; E-Mail: LHuniche@health.sdu.dk; 3 Department of Health Science and Technology, Faculty of Medicine, Aalborg University, Niels Jernes Vej 10, Aalborg DK-9920, Denmark; E-Mail: et@adm.aau.dk

**Keywords:** tele-rehabilitation, COPD patients, semi-structured interviews, qualitative methods, home tele-health

## Abstract

The aim of this paper is to describe patients’ attitudes towards tele-rehabilitation in the Danish TELEKAT (for Telehomecare, Chronic Patients and the Integrated Healthcare System) project, in order to better understand patients’ behavior when performing tele-rehabilitation activities in home surroundings. A total of 111 COPD patients were included in the study, and they were randomized into an intervention group (n = 60) and a control group (n = 51). However, a non-randomized design was used to analyze the qualitative perspectives of the patients’ attitudes towards tele-rehabilitation. From the intervention group, 22 COPD patients were selected for qualitative interviews and participant observation in their homes. The theoretical framework for this study is based on learning theory and the “communities of practice” approach inspired by Etienne Wenger. COPD patients exhibit four types of attitudes about their tele-rehabilitation: indifference, learning as part of situations in everyday life, feeling of security and motivation for performing physical training. The patients express the view that they circulate between these attitudes depending on their physical and emotional state as they perform their training. The COPD patients and healthcare professionals have created a community of tele-rehabilitation across sectors, exchanging experiences, stories and strategies for how to manage rehabilitation in home surroundings.

## 1. Introduction

It is estimated that 64 million people worldwide suffer from chronic obstructive pulmonary disease (COPD). In 2005, three million people died of COPD, equivalent to 5% of all deaths globally that year [1]. Patients with severe and very severe COPD experience disabling physical symptoms, especially breathlessness, as well as psychological distress, social isolation and co-morbidity [2,3,4,5]. Self-management education of COPD patients has been used to help them increase their physical activities and obtain more effective management of their disease. A Cochrane review of self-management education for COPD patients concludes that there is heterogeneity in the types of interventions, study populations, follow-up time, and outcome measures regarding the form and content of the self-management education programs for COPD patients [6]. A systematic review of home tele-health for COPD patients concludes that home tele-health was found to reduce rates of hospitalisation, emergency department visits and number of hospital bed days, but the results varied between the studies [7]. Self-management education has become an integral part of tele-rehabilitation programmes today. A systematic review has found only a small number of studies on tele-rehabilitation and that the benefits for COPD patients have not yet been confirmed [8]. Some common approaches to the self-management and tele-rehabilitation of chronic health conditions have been identified, but no specific approaches have been shown to be superior to others; rather, the various approaches are often complementary [9]. Self-management programmes require a multifaceted approach that incorporates not only teaching about various disease characteristics but also the implementation of strategies to change behavior in patients [10]. A review article describing daily life among COPD patients points out that treatment and rehabilitation of the acute exacerbation of COPD should focus on interventions that affect daily life activities [5]. The present study, based on the project “Telehomecare, Chronic Patients and the Integrated Healthcare System” (the TELEKAT project), has developed and tested a preventive tele-rehabilitation programme for COPD patients based on cooperation between hospital, district nurses, healthcare centre and general practitioners (GPs). This study focuses on those COPD patients who have been selected for self-monitoring and maintenance of rehabilitation activities in their homes in order to better manage their own disease and avoid readmission to hospital. Results [11] from the TELEKAT project show a significant decline in admission rate for those COPD patients (n=111) who have participated in the tele-rehabilitation programme. In this light, the aim of this paper is to describe patients’ attitudes towards tele-rehabilitation in home surroundings. The findings of the project are being used to develop more effective guidelines for selection and guidance of COPD patients in future tele-rehabilitation programmes. This paper addresses the following research question: What are COPD patients’ attitudes towards tele-rehabilitation as seen from a learning perspective?

The research question will be explored from the perspective of learning theory and the “communities of practice” approach, as inspired by Etienne Wenger (see section on theoretical framework). This suitability of learning theory and the “communities of practice” approach framework was based upon the TELEKAT projects focus on network cooperation between patients, relatives and healthcare professionals. 

## 2. Methods

### 2.1. Study Design

The case study method [12] was chosen as the overall research strategy for this study. A triangulation of data collection techniques has been used in order to provide multiple sources of evidence in the case study, such as document analysis, participant observation, and qualitative interviews with COPD patients and healthcare professionals. Data collection will be elaborated in the following sections.

### 2.2. Ethical Approval

Ethical approval was obtained from the local Ethics Committees (August 27, 2008/N-20080049). The study was conducted according to the Declaration of Helsinki. The project was reported to the Danish Data Protection Agency (7 August 2008). 

### 2.3. Participants Recruitment

In the TELEKAT project, a randomized study was conducted. However, a non-randomized design was used to elucidate the qualitative aspects of the patients’ attitudes towards tele-rehabilitation. The patients for the TELEKAT study were selected on the basis of the following inclusion and exclusion criteria (see Table 1). 

**Table 1:** Criteria for inclusion and exclusion of COPD patients in the TELEKAT project.

**Inclusion criteria**
Over 18 years Can understand oral and written trial information Diagnosed COPD in stages III & IV (severe and very severe COPD) COPD as primary cause of reduction in function
**Exclusion criteria**
Living outside Aalborg Municipality Heart disease that could limit physical function Mental illness Terminal malignant disease Severe rheumatoid arthritis Pregnancy

The COPD patients were recruited to the study by referral from doctors and nurses at a healthcare center, their general practitioner (GP) or the pulmonary hospital ward. After confirming eligibility and obtaining written informed consent, a nurse, blinded for patients, healthcare professionals and researchers, performed concealment. A total of 111 COPD patients were included in the study, and they were randomized into an intervention group (n = 60) and a control group (n = 51). A non-randomized design was used for analyzing the qualitative perspectives. The intervention group joined the tele-rehabilitation programme (see below). The control group, after inclusion in the study, received instructions on performing home exercises. The patients in the intervention and control groups were instructed on standardized information on exercises and received a set of instructional guidelines from the Danish Lung Association. The exercises included sitting exercises on a chair, exercises for the legs, stretching of neck muscles, standing exercises for arms and chest cavity and walking exercises. After receiving these instructions, they were responsible for performing the activities themselves, and they had no contact with the healthcare professionals who had instructed them in the home exercises. In order to identify COPD patients’ attitudes towards tele-rehabilitation, 22 COPD patients from the intervention group were selected for interviewing using the following criteria: men and women of different ages, civil status and educational backgrounds and according to the clinical setting where they were treated. The 22 interviewees were selected at random over two years and were recruited from the following clinical settings: (1) the local hospital, (2) the local healthcare centre, (3) the district nurses’ unit or (4) the participating GP’s clinics. Two researchers conducted the interviews. After interviewing 22 patients, we felt that we had reached the point of data saturation and no more new knowledge was forthcoming.

### 2.4. The Tele-Rehabilitation Programme

After the COPD patients were enrolled in the study, the doctor obtained baseline clinical data, and a nurse and doctor instructed each patient how often they had to measure values such as blood pressure, pulse, weight, oxygen level and lung function (spirometry) during a week. The COPD patients had a tele-health monitoring box installed in their home for a period of 16 weeks. On installing the CE-marked tele-health equipment (Mymedic/Mymedic plus from Tunstall Healthcare, Aalborg, Denmark) in the home, the patient or a relative was instructed on how to take the clinical measurements, how to use a step counter and how to perform the home-based exercises. In order to provide standardized information on the exercises, each patient was given a set of instructional guidelines from the Danish Lung Association. The exercises included sitting exercises on a chair, stretching of neck muscles, exercises for the legs, standing exercises for arms and chest cavity and walking exercises. The patients also performed exercises on their own. A calibration exercise was conducted with the patients and their relatives in order to avoid information bias.

Using wireless technology, the tele-health monitor (Mymedic/Mymedic plus) collected and transmitted data via a secure line. The data were sent to a web-based portal or to the patient’s electronic health record. Healthcare professionals such as the GP, district nurses, nurses and doctors at the healthcare centre or hospital could then assess the patient’s data, monitor the patient’s disease and training inputs and offer advice on development of symptoms, medication, exercises and general questions from the patient. The patients participating in the tele-rehabilitation programme had contact with healthcare professionals via e-mail or phone once or twice a week. If certain symptoms appeared, such as the onset of an exacerbation, the patients and healthcare professionals maintained daily contact. The patients and relatives assessed the data on the web portal and could decide with whom they wanted to share their data, but this was based on written consent. 

The patients had the opportunity to communicate with each other via a web portal and to exchange ideas or learning from each other on how to handle their disease in everyday life or to network with each other. Once per month, a tele-rehabilitation team consisting of health care professionals from primary and secondary care would conduct a video conference in order to coordinate their activities and discuss the COPD patients’ individual rehabilitation programmes. 

### 2.5. Data Collection

A triangulation of data collection techniques has been used in order to provide multiple sources of evidence [12] in the case study. The following data collection techniques were used.

#### 2.5.1. Documents

Documents were collected in order to obtain background knowledge and an understanding of the context for rehabilitation of COPD patients’ current rehabilitations programmes. Brochures from the healthcare centre and hospital also were studied by the researchers. 

#### 2.5.2. Participant Observation and Interviews in Patients’ Homes

The study was designed so that each patient (n = 22), during the 16 weeks in which they were enrolled in the tele-rehabilitation programme, was visited three times in their home by a researcher. The visits took place during the first week after the patient had been enrolled in the programme, eight weeks into the programme, and one week after the equipment had been removed from their homes. The visits were carried out by two researchers: the first author (n = 15) and the second author (n = 7). The same researcher visited the same patient for all three visits. The interviews were recorded on a digital recorder with prior informed consent by the patient.

The goals of the visits were two-fold. First, as a form of participant observation, inspired by Delamont [13], the visits enabled the researcher to obtain a basic understanding of how the patients performed exercises in their home. Patients were observed measuring values, communicating with healthcare professionals on rehabilitation issues, performing their exercises, and communicating with family members. The participant-observation visits lasted 30–60 min. The second aim of the home visits was to conduct qualitative interviews, inspired by Kvale [14], and to obtain an in-depth understanding of the patients’ attitudes and experiences as participants in the tele-rehabilitation programme. Interviews were conducted at each visit and lasted 1–1.5 h. Semi-structured interview guides were used. All interviews were transcribed, and two researchers conducted the interviews. Nineteen patients were visited three times, and three patients had only two visits due to their being admitted to the hospital prior to the third visit.

Patients were asked the following questions: What significance has tele-rehabilitation had for your management of your disease? Has the tele-rehabilitation been important in relation to your own training efforts? What has it meant to you concretely in relation to how you manage exacerbations, administer medicine, use of oxygen, *etc.*? 

#### 2.5.3. Interviews with Healthcare Professionals

The patients were referred to the tele-rehabilitation programme from a hospital, district nurse, healthcare centre or general practitioner (GP). The healthcare professionals supervised the patients’ entry into the tele-rehabilitation programme, so we found it relevant to interview them also. Semi-structured interviews were conducted with representatives from five healthcare professions (see Table 2). The interviews lasted about one hour and were conducted by two researchers and then transcribed. Each of the five groups of healthcare professionals had been in contact with one or more of the COPD patients who followed the TELEKAT tele-rehabilitation program. The first author conducted all the interviews with the healthcare professionals together with a research colleague. The interviews were recorded on a digital recorder with prior informed consent by the healthcare worker.

Healthcare professionals were asked questions such as: How did the COPD patients react to having to measure their own values? How would you describe the patients’ reaction to measuring their own data? How would you characterize the patients’ attitudes toward tele-rehabilitation? 

**Table 2:** Occupations of healthcare professionals interviewed.

Occupation of respondent	Number interviewed
GPs	6
Nurses at the hospital	4
Doctors at the hospital	2
Nurses at the healthcare centre	6
District nurses	8
Total	26

### 2.6. Theoretical Framework

The theoretical framework for this study is based on learning theory and the “communities of practice” approach, as inspired by Etienne Wenger [15,16]. Wenger has defined “communities of practice” as groups of people who share a concern or passion for something they do and who learn how to do it better as they interact regularly*.* Wenger sees learning as a social practice centered around knowledge-sharing. According to Wenger [15], a learning process is more than an individual cognitive process. Learning takes place in interaction with others, with whom one has a common interest. Hence, one becomes a part of a social learning process. Through the communities of practice, the participants gained more knowledge and understanding of their common interest. 

Three elements distinguish a community of practice from other kinds of groups and communities [16]. 

**The domain:** Participants in a community of practice comprise more than a network of people. The participants share a domain of interest and a commitment to the domain; in this case, the domain is the tele-rehabilitation of the COPD patients.

**The community**: Participants in the domain are involved in joint activities and discussions, share information and build relationships that enable them to learn from each other and help each other. The community in the TELEKAT project consists of COPD patients, relatives and healthcare professionals. 

**The practice**: According to Wenger, participants in a community of practice are “practitioners”. Over time and in sustained interaction, the participants develop a shared practice and repertoire of resources: they exchange experiences, stories, tools, and techniques for addressing recurring problems, here in connection with tele-rehabilitation issues. In the TELEKAT project, the practice is constituted by COPD patients, relatives and healthcare professionals.

A community of practice thus involves much more than the technical knowledge or skill associated with undertaking some task. Participants will be involved in a set of relationships over time. The communities of practice will be organized around some particular area of knowledge and activity that endows participants with a sense of joint enterprise and identity. In order for a community of practice to function, it needs to generate and appropriate a shared repertoire of ideas, commitments and memories. The mutual interactions among members in the TELEKAT project can be viewed in from a community of practice perspective. The community is bound together and the engagement and joint practices help facilitate the kinds of relationships of trust on which communities are built and maintained. This community of practice approach is better than other theoretical approaches as it clear up how technology can facilitate and support learning processes and network among participants. 

### 2.7. Data Analysis

All the transcribed interviews from patients and healthcare professionals were coded with Nvivo 8.0 software and analyzed in steps inspired by Kvale [14]. The data were analyzed using a combination of deductive and inductive strategies. The code tree was formed on the basis of key definitions and concepts (*in vitro* nodes) from the theoretical framework and from interviews (*in vivo* nodes). When formulating the concepts from the respondents, qualitative interviews were studied and coded on the basis of initial impression. The next step was a rough coding and refined coding on the basis of reviews of coded data and adjustments. This step sought to identify key topics and patterns relevant to identifying categories of attitudes of patients on tele-rehabilitation. This latter phase included an in-depth interpretation contrasted with the participants’ common-sense understanding. In this phase, the interviews were analyzed in order to identify participants’ motivations and perceptions. The coding and analysis was carried out by the first two researchers, both of whom have backgrounds in nursing, organisational development and psychology. To ensure intercoder reliability, the same two researchers initially coded two interviews each, compared codes and agreed on definitions for subsequent coding.

## 3. Results

### 3.1. Domain

All patients interviewed (n = 22) expressed commitment to the domain of tele-rehabilitation. They had a common interest in learning about how to utilise new technology to monitor themselves and perform rehabilitation activities in home surroundings. One patient states:

“*It is interesting to learn to use the technology and to monitor myself.*”

The healthcare professionals experienced the patients as having a common interest in tele-rehabilitation and in participating directly in their rehabilitation.

“*All patients included in the study were very eager to participate in testing the new technology.*”

### 3.2. Community

Of the 22 patients interviewed for this study, 17 reported having developed a relationship based upon trust and mutual understanding of rehabilitation in home surroundings. These 17 patients expressed the view that the technological platform in the TELEKAT project opened the possibility for them to obtain data, share data and communicate with healthcare professionals and other patients independently of time and space. Twelve patients reported that they gained new knowledge by communicating and interacting about the measured values, symptoms, medication and exercises as well as in the social and cultural process of exchanging experiences, stories from everyday life and how to address rehabilitation-related issues in their home surroundings. As one patient stated:

“*My weekly contact with the healthcare professionals helped me to gain new knowledge about how to handle my own disease in different situations of everyday life.*”

Another patient explained:

“*For me as a patient, it has been important to get to know other COPD patients and to hear about their symptoms and experiences about how they live with their disease in everyday life.*”

In the view of the healthcare professionals, the COPD patients were seen as having obtained a feeling of being cared for when participating in the TELEKAT tele-rehabilitation programme, thus reducing their feelings of isolation. A healthcare professional explained:

“*The patients enjoyed being able to share experiences with us as healthcare professionals and with the other patients.*”

### 3.3. Practice Indifference

A small number of patients (5/22) experienced indifference toward the tele-rehabilitation measures. The patients argued that is was because the measured values (blood pressure, pulse, weight, spirometry and saturation) were stable. These patients reported that they were unable to observe any connection between measured values and physical training over time when they followed their data on the TELEKAT web portal or on their tele-health monitor. The patients stated that it did not make sense to measure values while doing the exercises at home. One COPD patient said:

“*I don*’*t feel that measuring my values makes a difference for me—They are stable all the time.*”

The healthcare professionals have identified the same attitude among those patients whose measured values were stable over time. In the words of one healthcare professional*:*

“*COPD patients who measure stable values do not benefit from doing preventive tele-rehabilitation.*”

### 3.4. Learning as Part of Situations in Everyday Life

Twelve of the 22 patients interviewed regarded the tele-rehabilitation programme as a learning process. They were learning how to be better at integrating physical training into their everyday lives and to better manage their chronic disease. 

These 12 patients found that being enrolled for 16 weeks in the tele-rehabilitation programme gave them time to try new exercises, to become more involved and to adjust their training program to their home environment and situations in everyday life. These patients regarded the tele-rehabilitation programme as a social and learning process which included the family and network of the COPD patients. The patients reported that family and network became more engaged in the tele-rehabilitation program of helping the patient to integrate the activities into their everyday routine and maintain the focus on exercise as a normal part of their everyday lives. Over time, they saw a change in their attitude toward physical exercise. The patients stated that they became more aware and reflected upon the measured values and symptoms in their COPD. Via their interaction with the healthcare professionals and other patients in the programme, they learned to become more aware of their own symptoms and to know when it was necessary to contact a doctor at an early stage in order to seek treatment. As one patient said:

“*Seeing my data on the web portal gives me a better understanding of how to exercise and interpret the development of my symptoms when I experience the onset of an exacerbation*.*”*

The healthcare professionals reported that they gave some of the patients more responsibility for managing their own disease, and the patients received a treatment plan consisting of prescriptions for penicillin and hormones and guidelines for what to do in case symptoms appeared. In this way, the patients became more active, changing their mind-set, and were able to perform self-management of their COPD. A doctor at the hospital remarked:

“*We found that the measured values that were accessible and visualised through graphics provided the patients with an overview of the development of their own symptoms, and they learn to act and foresee an exacerbation.*”

### 3.5. Feeling of Security

Five of the 22 patients interviewed expressed the view that contact with the healthcare professionals gave them a feeling of security. Those patients who were using oxygen in their homes felt that the 16-week tele-rehabilitation period was too short. They preferred the possibility of being monitored permanently. 

Healthcare professionals monitored the values, and the patients were contacted if the values exceeded normal range. In cases where symptoms developed, e.g., an exacerbation, the healthcare professionals would remain in daily contact with the patients via phone or e-mail consultation and could initiate treatment by prescribing penicillin or hormones so that the patient could avoid hospitalization. Hence, one COPD patient said:

“*I feel safe by measuring my saturation and knowing that the nurse at the hospital can see my data.*”

Healthcare professionals expressed the view that the COPD patients using permanent oxygen had a feeling of security within the tele-rehabilitation programme:

“*COPD patients who use oxygen every day benefit from being able to monitor their saturation and from being able to ask us for advice.*”

### 3.6. Motivation

Fifteen of the 22 patients interviewed expressed the view that they became motivated on performing physical training and joining the tele-rehabilitation program. These patients were encouraged by several aspects of the TELEKAT programme: 

First, they found it convenient that they could do their exercises at home, at any time. Second, they were encouraged by having access to remotely supervised feedback from healthcare professionals. Third, the patients stated that interaction with healthcare professionals and other patients encouraged them to carry out their physical training at home and to push themselves. Fourth, being able to actually see the graphically presented data (blood pressure, pulse, weight, spiometry and saturation) on the web portal or tele-health monitor motivated the patients to continue training and to compete with themselves, especially when the measured values showed improvement over time. A COPD patient said:

“*I get motivated when I see my data on the web portal ... It is a milestone*,*and I want to improve my values by exercising*.”

The healthcare professionals viewed themselves as the patients’ coaches in the tele-rehabilitation programme. A healthcare worker explained:

“*I feel that the COPD patients are getting to be more active and motivated to do training at home. I feel like a coach for them.*”

### 3.7 Changing Attitudes toward Tele-rehabilitation

The patients stated that in practicing tele-rehabilitation, their feelings alternated between indifference, learning, a feeling of security and motivation when proceeding with their training and that they alternated between the themes. Some patients have articulated more than one topic on tele-rehabilitation. One patient states:

“*My disease is like a roller coaster. I have my ups and downs, so my attitudes on rehabilitation can vary depending on the status of my disease.*”

Table 3 presents the key characteristics of the patients who participated in the study. 

**Table 3:** Characteristics of patients interviewed in the TELEKAT study in interquartile range (IQR).

Variable	Males (n = 8)	Females (n = 14)
Age in years (IQR)	69.4 (64; 73.8)	66.4 (44.6; 81.1)
No. married/co-habiting	3	4
No. living alone	5	10
No. employed	0	2
Retired	8	12
Smoker	2	2
Former smoker	6	12
Using oxygen at home	0	2
Forced expiratory volume in 1 sec, in litres (FEV1) (IQR)	0.97 (0.11; 1.41)	0.81 (0.36; 1.34)
Weight in kg (IQR)	85.74 (50.8; 108.1)	70.23 (50; 85.9)
Body mass index in kg/m^2 ^(IQR)	28.4 (18.6; 35.7)	26.36 (20.2; 34)
Oxygen saturation (% on ambient air)	92.5 (90; 95)	93.3 (91;96)
Blood pressure in mmHg (IQR)	142/83 (107/80; 180/90)	134/81 (97/52; 178/104)
Heart rate in minutes (IQR)	83 (70; 106)	88 (73; 111)
MRC dyspnoea score (IQR)	3.4 (2; 5)	3.72 (3; 5)

Table 4 presents major themes in COPD patients’ attitudes towards tele-rehabilitation according to the three main elements of Wenger´s community of practice framework: domain, community and practice. 

**Table 4:** Themes in COPD patients’ attitudes towards tele-rehabilitation.

	COPD patients’ attitudes toward tele-rehabilitation	Healthcare professionals’ assessment of COPD patients’ attitudes towards tele-rehabilitation
**Domain**	Commitment Joint interest in rehabilitation	Interested in tele-rehabilitation
**Community**	Development of relationships with healthcare professionals Development of mutual understanding of rehabilitation in home surroundings Gaining new knowledge on own disease by sharing data	Feeling looked after Reduced feelings of isolation
**Practice/**	Indifference Learning as a part of situations in everyday life Feeling of security Motivation	Patients do not engage in own treatment Active participation in own disease management Feeling of security Patients are changing mindset on rehabilitation and change behavior on handling own disease

## 4. Discussion

Based upon Wenger’s theory of learning and the social context of “community of practice”, the COPD patients’ attitudes towards tele-rehabilitation have been identified. The COPD patients expressed a general commitment and interest in the domain of tele-rehabilitation. However, when they became part of a community and in practicing tele-rehabilitation on a daily basis, an enhanced perspective developed. 

The interaction between the COPD patients and healthcare professionals in the tele-rehabilitation programme can be characterized in terms of Wenger’s approach as a “community of tele-rehabilitation”. This community links COPD patients, their family members and healthcare professionals across sectors. The COPD patients have expressed the view that their relationships with the healthcare professionals had developed from that of being subordinated to professional authority to a relationship of dialogue, where the focus was on mutual learning [17]. 

The interaction between patients and healthcare professionals catalysed the rehabilitation process so that it became more than an individual cognitive process centred on the patient. The learning was also distributed amongst the family and network of the COPD patients. One study has emphasized that the support of families and network is important if COPD patients are to maintain lifestyle changes over time [18,19]. We have not been able to identify any studies that focus on patients’ attitudes toward tele-rehabilitation and learning.

The majority of the COPD patients expressed the view that the technological platform in the TELEKAT project facilitated communication and knowledge-sharing between patients and healthcare professionals. Seen from the perspective of Wenger´s theory, the tele-rehabilitation programme has facilitated social learning processes for the benefit of patients, relatives and healthcare professionals. 

In terms of Wenger’s theory, we have shown that COPD patients exhibit four attitudes about performing tele-rehabilitation: indifference, learning as part of situations in everyday life, feeling of security and motivation. The patients state that they alternated between these attitudes depending on how they were proceeding with their training and disease. Over the 16-week test period, the COPD patients showed changing attitudes about their participation in the tele-rehabilitation programme. One patient viewed her participation as a disease roller coaster. 

A review article on the lives of COPD patients states that [5]: “The gap between what a patient is capable of doing and what he is doing in the daily life can be significant: this can possibly be addressed though self-management education. Not only should intervention on a physiological level be incorporated, but also interventions that are effective in achieving behaviour change. The identified attitudes and other results [11] in the TELEKAT project indicate that the COPD patients in our study have experienced a change in their behaviour. They became more aware of developments of their own symptoms and contacted a physician at an earlier stage than they did prior to entering the programme.

Healthcare professionals and patients expressed the view that the design and function of the web portal promoted networking between the parties. Observations and qualitative analysis showed that being able to see the measured and visualized values on the screen gave them a better understanding of their own disease, thus motivating them to involve themselves more deeply in their rehabilitation activities. This tendency has been seen in studies of home monitoring of other chronic diseases [20]. In the TELEKAT study, however, the patients articulated the view that they felt well cared for and were secure in the knowledge that the healthcare professionals were there for them “at the end of the line”. This made them feel more secure when carrying out their home-based rehabilitation activities, despite the fact that no one was physically present to supervise them. The patients truly “never felt alone”.

A qualitative study has described severe COPD as “a way of life” rather than simply an illness that disrupts life [21]. Over the years, the COPD lifestyle becomes familiar for the family and network. The findings of this study, as well as other studies [9,22], challenge the current assumptions about how to plan rehabilitation activities for patients suffering from severe and very severe COPD. Understanding how COPD patients approach their tele-rehabilitation can provide knowledge to improve the content of future tele-rehabilitation programmes, and knowledge about how to identify and stratify COPD patients in tele-rehabilitation programmes. Recall that there was a group of COPD patients in our study who were indifferent to the tele-rehabilitation program. We need to learn to identify those patients who will benefit from tele-rehabilitation and to design appropriate stratifications tools to cull out those patients who do not need tele-rehabilitation. We have not been able to identify any studies on how to stratify patients for tele-rehabilitation. More research is needed in this area.

A key issue for any case study is that of generalizability. In order to optimize generalization of case studies, reference literature [23] recommends strategic case selection or analytical generalization. We shall not venture into this discussion here, but merely point out that in the TELEKAT project, analytical generalization has been applied by using a theoretical framework proposed by Wenger and a triangulation of data collection. The analysis presented here supports the process of analytical generalization. By using the case study and an analytical generalisation of data, we have identified attitudes about tele-rehabilitation that are specific to COPD patients. Are the attitudes about tele-rehabilitation expressed by the COPD patients specific to this group? Or might they also be typical of other patients groups? More research is needed to address this issue. 

Our findings regarding variations in COPD patients’ attitudes towards tele-rehabilitation can be used for developing stratification tools for including patients in tele-rehabilitations programmes in the future and for planning more individual tele-rehabilitation programmes for the COPD patients. 

The limitation of the study is that it uses a small sample. We have not interviewed the COPD patients in the control group, a procedure which could have been helpful for comparison with the patients in the intervention group. We have also not included the variable of frequency: *i.e.*, how many times per day or week the patients measure their values. In this connection, one might hypothesize that the more a COPD patient measures their values, the greater engagement the patient has in the project. 

## 5. Conclusions

COPD patients exhibit four attitudes about their tele-rehabilitation: indifference, learning as part of situations in everyday life, feeling of security and motivation to perform physical training. The patients express the view that they alternate between these attitudes, depending on their physical and emotional state as they perform their rehabilitation exercises. A community of tele-rehabilitation across sectors has been created between the COPD patients and COPD patients and healthcare professionals. The TELEKAT community is characterised by mutual learning and by the emergence of new relations between the participants focusing on strategies for managing rehabilitation in home surroundings. We need to learn to identify those patients who will benefit most from tele-rehabilitation and to design appropriate stratifications tools so that we can find precisely those patients for whom tele-rehabilitation will be most effective.
